# Effect of paternal age on offspring birth defects: a systematic review and meta-analysis

**DOI:** 10.18632/aging.104141

**Published:** 2020-11-20

**Authors:** Yiwei Fang, Yongfeng Wang, Meilin Peng, Jia Xu, Zunpan Fan, Chunyan Liu, Kai Zhao, Huiping Zhang

**Affiliations:** 1 Institute of Reproductive Health, Tongji Medical College, Huazhong University of Science and Technology, Wuhan, China

**Keywords:** paternal age, old father, young father, birth defect, congenital abnormality

## Abstract

Objective: This systematic review and meta-analysis was aimed at determining whether paternal age is a risk factor for offspring birth defects.

Results: A total of 38 and 11 studies were included in the systematic review and meta-analysis, respectively. Compared with reference, fathers aged 25 to 29, young fathers (< 20 years) could increase the risk of urogenital abnormalities (OR: 1.50, 95 % CI: 1.03–2.19) and chromosome disorders (OR: 1.38, 95 % CI: 1.12–1.52) in their offsprings; old fathers (≥ 40 years) could increase the risk of cardiovascular abnormalities (OR: 1.10, 95 % CI: 1.01–1.20), facial deformities (OR: 1.08, 95 % CI: 1.00–1.17), urogenital abnormalities (OR: 1.28, 95 % CI: 1.07–1.52), and chromosome disorders (OR: 1.30, 95 % CI: 1.12–1.52).

Conclusions: Our study indicated that paternal age is associated with a moderate increase in the incidence of urogenital and cardiovascular abnormalities, facial deformities, and chromosome disorders.

Methods: PubMed, Web of Science, the Cochrane Library, and Embase were searched for relevant literatures from 1960 to February 2020. The systematic review follows PRISMA guidelines. Relevant meta-analyses were performed.

## INTRODUCTION

Previous study has shown that the overall prevalence of birth defects in live births ranges from 3–5 % [1]. Birth defects are a major cause of perinatal mortality, accounting for more than 20 % of infant deaths [2, 3]. Moreover, birth defects are one of the strongest known risk factors for childhood cancers [4], causing serious effects in children’s health; such defects are also associated with high risks of preterm birth (PTB), low birth weight (LBW), and infant death [5]. Thus, birth defects do not only increase medical burden but also place an economic burden on families and society [6].

According to Medical Subject Headings (MeSH) of the PubMed database, classification of urogenital abnormalities, digestive system abnormalities, nervous system malformations, cardiovascular abnormalities, facial deformities, musculoskeletal abnormalities, and chromosome disorders are shown in Supplementary Table 1.

In accordance with a consensus, young fathers are aged 20 years and below, whereas older fathers are older than 40 years [7]. In developed regions, such as Europe and the USA, the proportion of late marriage and childbirth is increasing [7, 8], whereas early marriage and early childbearing are on the ascendancy in developing countries [9, 10]. Some studies have assessed the relationship between paternal age and birth defects in offspring, but no substantive conclusion, even contradictory has been drawn [10–12]. In previous years, a few systematic reviews and meta-analyses have evaluated the relationship between paternal age (especially advanced paternal age) and birth defects, including congenital heart disease, cleft lip and palate, neural tube defects, gastroschisis, and trisomy 21 syndrome. However, these studies did not include other birth defects, such as hydranencephaly of common nervous system malformations and trisomy 13 and trisomy 18 syndromes of common chromosome disorders. Moreover, systematic reviews and meta-analyses about urogenital abnormalities and digestive system abnormalities seem limited [11, 13, 14]. Therefore, a further systematic review and meta-analysis of birth defects is needed to fill the gap [15–17].

This systematic review and meta-analysis focused on the influence of paternal age, particularly of old (> 40 years old) and young fathers (< 20 years old), on offspring birth defects in each system and chromosomal abnormalities. This meta-analysis is the first of its kind that focuses on the effect of paternal age on urogenital and digestive system abnormalities; the results of this work contributes to a comprehensive understanding of the risk factors for birth defects and its effective prevention.

## RESULTS

### Study selection and characteristics

**Figure 1:**
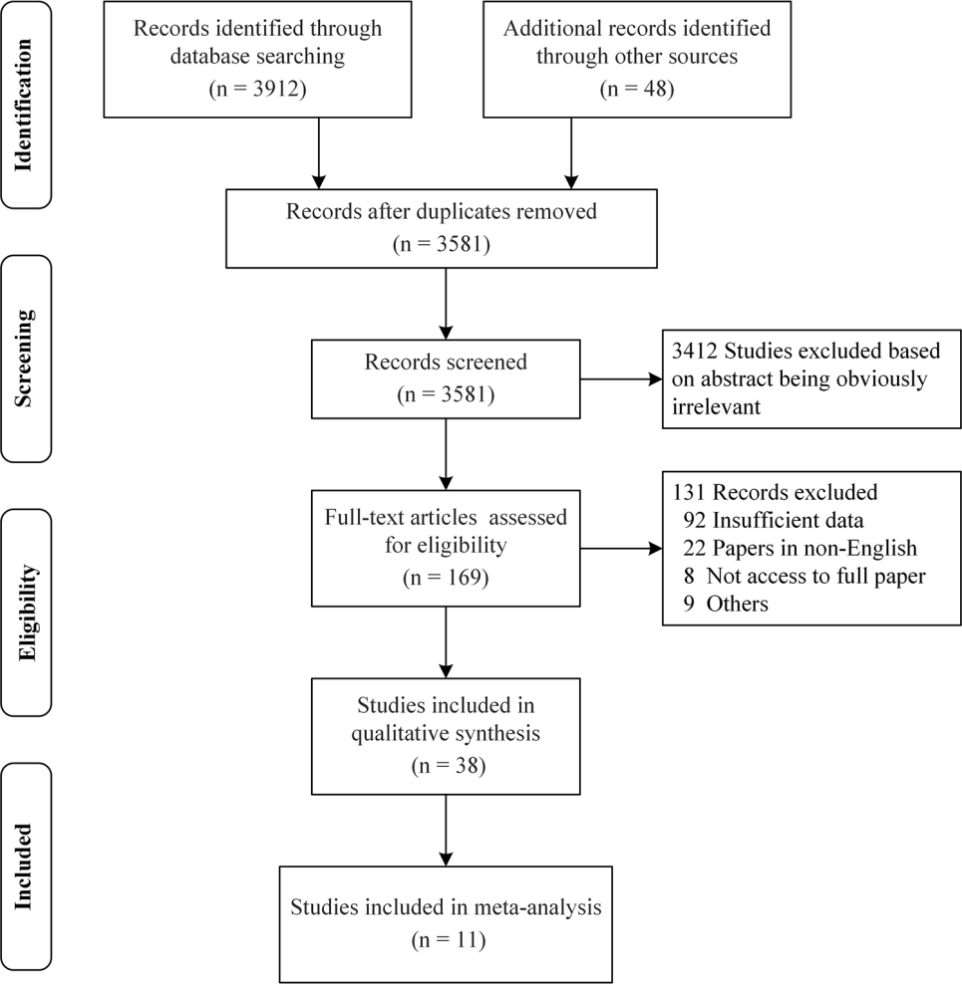
**PRISMA flow diagram for a systematic review and meta-analysis.** A total of 3581 articles were identified after duplicates removed. Out of the 3581 articles, 3412 articles were directly excluded after reading the titles and abstracts, and 131 articles were excluded for some reasons after reading the full text. Finally, 38 and 11 studies were included in the systematic evaluation and meta-analysis, respectively.

We identified a total of 3581 articles published between 1962 and 2020 after duplicates were removed. A total of 3412 articles were directly excluded after reading the titles and abstracts, and 131 articles were excluded after reading the full text for the following reasons: insufficient data (92), non-English (22), no access to the full paper (8), and others (9) (Figure 1). Lastly, a total of 38 and 11 studies were included in the systematic evaluation and meta-analysis, respectively. Figure 1, Table 1 and Supplementary Table 2 respectively show the process of literature inclusion and summarize the characteristics of the included literature.

**Table 1:** Summary results of the systematic review of the association between young and old father and birth defects.

**Birth defects (Numbers of studies)**	**Risk**	**Number of studies**		**Total number of cases**
		**Young paternal age (<20 years)**	**Advanced paternal age (≥40 years)**	
Urogenital Abnormalities (6)				5217
	Increased	1	2	
	Decreased	0	0	
Digestive System Abnormalities (5)				5823
	Increased	0	0	
	Decreased	0	1	
Nervous System Malformations (9)				8191
	Increased	1	4	
	Decreased	0	0	
Cardiovascular Abnormalities (11)				32190
	Increased	2	6	
	Decreased	1	1	
Facial deformity (13)				18807
	Increased	1	4	
	Decreased	0	1	
Musculoskeletal Abnormalities (14)				31479
	Increased	3	3	
	Decreased	1	3	
Chromosome Disorders (10)				18108
	Increased	2	4	
	Decreased	0	2	

Of the 38 studies, 18 were case-control studies, and the rest were cohort studies. In accordance with the NOS, the quality of research was evaluated. The following were obtained: 27 high-quality studies (NOS scores ≥ 7), nine medium-quality studies (NOS scores of 5–6), and two low-quality studies (NOS scores ≤ 4). Twenty-six studies adjusted or controlled for maternal age, and 12 did not.

The number of reported cases is as follows: more than 1,000 in 14 studies; 101-1,000 in 19 studies, and not more than 100 in five studies. The number of studies conducted by region is as follows: 15 in North America (12 in the USA), 15 in Europe (five in Norway and four in Denmark), six in Asia (three in China), two in South America, and only one in Africa. One study was conducted simultaneously in the United States and the Czech Republic. Twenty-seven studies began before 2000, and nine studies were conducted after 2000; 31 studies lasted longer than 3 years, and five studies lasted less than 3 years. The longest study lasted for more than 40 years, whereas the shortest study lasted for less than one year. Two studies did not report the time of study execution.

Regarding the assessment of exposure factors (paternal age), 32 studies clarified the paternal age. However, different studies used various methods for categorizing paternal age; the most common categorization of paternal age was < 20, 20 – 24, 25 – 29, 30 – 34, 35 – 39, and > 39 years. In the present study, we defined fathers younger than 20 years as young fathers, older than 40 years as old fathers, and 25 – 29 years as the reference group.

### Meta-analytic results for birth defects in each system

### Urogenital abnormalities

**Figure 2:**
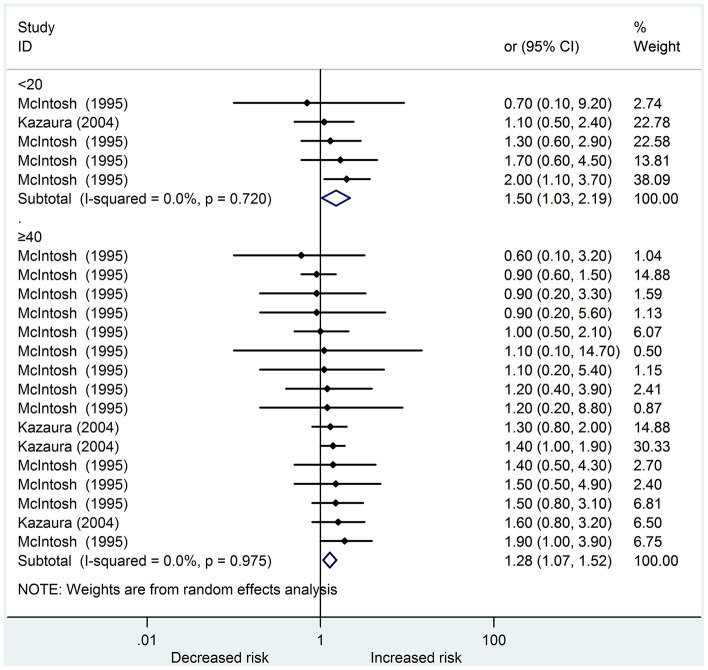
**Forest plot presenting the effect of young and old father on urogenital abnormalities in their offspring: Only two studies could be included in the meta-analysis.** Both young and old father increased the risk of urogenital abnormalities in offspring (OR 1.50, 95%CI 1.03-2.19; OR 1.28, 95%CI 1.07-1.52, respectively). There was no heterogeneity in these two subgroups (I^2^=0.0%, 0.0%, respectively) amongst the studies.

We identified six studies [14, 15, 18–21] that reported a total of 5217 cases about urogenital abnormalities in the systematic review; four were cohort studies, whereas two were case-control studies. On the basis of the NOS scoring criteria, five high-quality and one medium-quality studies were identified. In the meta-analysis, only two studies [14, 19] could be included. Young and old fathers increased the risk of offspring urogenital abnormalities (OR: 1.50, 95 % CI: 1.03 – 2.19; OR: 1.28, 95 % CI: 1.07 – 1.52, respectively). Among the studies, no heterogeneity was found in these two subgroups (I^2^ = 0.0 %, 0.0 %, respectively) (Figure 2). The results of the funnel plots and Egger’s test (P = 0.369) revealed no significant publication bias.

### Digestive system abnormalities

**Figure 3:**
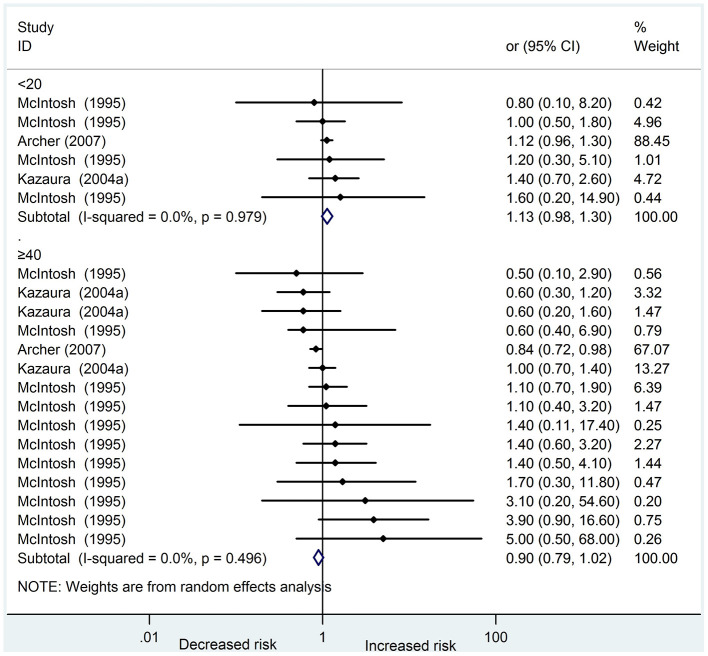
**Forest plot presenting the effect of young and old father on digestive system abnormalities in their offspring: Three studies were included in the meta-analysis.** The pooled OR in subgroup of young fathers and old fathers was 1.13(95%CI 0.98-1.30) and 0.90(95%CI 0.79-1.02), respectively. There was no heterogeneity in these two subgroups (I^2^=0.0%, 0.0%, respectively) amongst the studies.

Five studies [14, 15, 19, 20, 22] reporting a total of 5823 cases analyzed the association between paternal age and digestive system abnormalities in offspring. Of these studies, four were cohort studies, and one was a case-control study. In addition, all the studies were of high quality and adjusted for maternal age. Three studies [14, 19, 22] were included in the meta-analysis. The pooled ORs in the subgroup of young and old fathers were 1.13 (95 % CI: 0.98 – 1.30) and 0.90 (95 % CI 0.79 – 1.02), respectively. Among the studies, no heterogeneity was found in these two subgroups (I^2^ = 0.0 %, 0.0 %, respectively) (Figure 3). The results of the funnel plots and Egger’s test (P = 0.244) revealed no significant publication bias.

### Nervous system malformations

Nine papers [14, 15, 19–25] reporting a total of 8191 cases of nervous system malformations were included in the systematic review; seven were cohort studies, and two were case-control studies. In accordance with the NOS scoring criteria, eight high-quality and one medium-quality studies were identified. Five studies [14, 19, 22–24] were included in the meta-analysis. The pooled ORs in the subgroup of young and old fathers were 1.23 (95 % CI: 0.94 – 1.60) and 1.12 (95 % CI: 0.97 – 1.30), respectively. Among the studies, minimal heterogeneities were found in the two subgroups (I^2^ = 36.5 %, 33.5 %, respectively) (Supplementary Figure 1). The results of the funnel plots and Egger’s test (P = 0.071) revealed no significant publication bias.

### Cardiovascular abnormalities

Five cohort studies and six case-control studies [15, 19, 21, 22, 26–32] reported the association between paternal age and cardiovascular abnormalities; nine high-quality and two medium-quality studies were identified. The total number of cases of cardiovascular system malformations was 32,190. A meta-analysis of the data based on four studies [19, 22, 30, 31] showed that compared with fathers aged 25 – 29, younger fathers (< 20 years) did not increase the risk of cardiovascular abnormalities in their children, whereas older fathers (≥ 40 years) did. The pooled ORs were 1.05 (95 % CI: 0.96 – 1.16) and 1.10 (95 % CI: 1.01 – 1.20), respectively. Among the studies, minimal heterogeneity was found in the two subgroups (I^2^ = 2.1%, 37.6%, respectively) (Supplementary Figure 2). The results of the funnel plots and Egger’s test (P = 0.128) revealed no publication bias.

### Facial deformities

Thirteen papers [12, 14, 15, 19–22, 33–38], including nine cohort studies and four case-control studies, concentrated on paternal age as a risk factor for facial deformities in offspring; eleven were of high quality, one was of medium quality, and one was of low quality. A total of 18807 cases of facial deformities (most of the cleft lip or palate) were explored in thirteen studies. Older fathers (≥ 40 years) slightly increase the risk of facial deformities in their children, whereas younger fathers (< 20 years) did not. The pooled ORs were 1.08 (95 % CI: 1.00 – 1.17) and 1.14 (95 % CI: 0.99 – 1.31), respectively. Among the studies, no heterogeneity was found in the two subgroups (I^2^ = 0.0 %, 0.0 %, respectively) [14, 19, 22, 33] (Supplementary Figure 3). The results of the funnel plots and Egger’s test (P = 0.186) revealed no significant publication bias.

### Musculoskeletal Abnormalities

Thirteen papers [14, 15, 19–22, 39–45], including nine cohort studies and four case-control studies, concentrated on paternal age as a risk factor for musculoskeletal abnormalities in offspring; ten were of high quality, two were of medium quality, and one was of low quality. A total of 27546 cases of musculoskeletal abnormalities in fourteen studies were included in the systematic review. We included four studies [14, 19, 22, 44] in the meta-analysis, and the results showed that compared with fathers aged 25 – 29, younger (< 20 years) and older fathers (≥ 40 years) did not increase the risk of musculoskeletal abnormalities in offspring. The pooled ORs were 1.15 (95 % CI: 0.86 – 1.54) and 1.19 (95 % CI: 0.99 – 1.42), respectively (Supplementary Figure 4). Among the studies, medium heterogeneity was found in the two subgroups (I^2^ = 53.7 % and 41.4 %, respectively). The funnel plots and Egger’s test (P = 0.004) revealed a significant publication bias. We detected publication bias in the subgroup of young and old fathers. The Egger’s test found no publication bias in the subgroup of young fathers (P = 0.586) but found such bias in the subgroup of old fathers (P = 0.002). In addition, after correcting publication bias in the subgroup of old fathers via the nonparametric trim-and-fill method, the pooled OR was still not statistically significant (OR: 1.039, 95 % CI: 0.841 – 1.284). The funnel plots after correcting the publication bias in the subgroup of old fathers is shown in Supplementary Figure 5.

### Chromosome disorders

Ten papers [14, 15, 17, 22, 46–51], including five cohort studies and five case-control studies, were identified. Among them, eight papers were of high quality, and two were of medium quality. A total of 18108 cases of chromosome disorders, such as Trisomy 21, Trisomy 13, and Trisomy 18, were accessed in this systematic review. We conducted meta-analysis on four studies [14, 22, 46, 50] and revealed a moderately high risk of chromosome disorders in newborns of young and old fathers (OR: 1.38, 95 % CI: 1.01 – 1.89; OR: 1.30, 95 % CI: 1.12 – 1.52, respectively) in comparison with the reference fathers (25 – 29 years) (Supplementary Figure 6). Among the studies, medium heterogeneity was found in the two subgroups (I^2^ = 52.6 % and 62.1% for young and old fathers, respectively). The funnel plots and Egger’s test (P = 0.376) revealed no significant publication bias.

## DISCUSSION

Previously, a few works involving meta-analysis evaluated the association between paternal age and birth defects; however, most of them explored birth defects in general, without sorting the defects by systems. Based on the results of our meta-analysis, young fathers (< 20 years) could increase the risk of urogenital abnormalities and chromosome disorders in their offspring, whereas old fathers (≥ 40 years) could increase the risk of cardiovascular abnormalities, facial deformities, urogenital abnormalities, and chromosome disorders in their offspring. However, no significant difference was found between fathers younger than 20 or older than 40 in the incidence of musculoskeletal malformations, nervous system malformations, and digestive malformations in their offspring in comparison with fathers aged 25 – 29. Overall, the heterogeneities of the studies we included were small. Although, publication bias was found in the literature of skeletal musculoskeletal birth defects, the original conclusion did not change after correction by the nonparametric trim-and-fill method. Table 2 summarizes the main findings of the meta-analysis.

**Table 2:** Summary results of the meta-analyses of the association between young and old father and birth defects.

**Birth defects**	**Paternal age(years)**	**Pooled estimate (with 95% CI)**	**Heterogeneity (I^2^)**	**Publication bias**
Urogenital Abnormalities				NO
	<20	1.50 (1.03-2.19)	0.0%	
	≥40	1.28 (1.07-1.52)	0.0%	
Digestive System Abnormalities				NO
	<20	1.13(0.98-1.30)	0.0%	
	≥40	0.90(0.79-1.02)	0.0%	
Nervous System Malformations				NO
	<20	1.23(0.94-1.60)	36.5%	
	≥40	1.12(0.97-1.30)	33.5%	
Cardiovascular Abnormalities				NO
	<20	1.05 (0.96-1.16)	2.1%	
	≥40	1.10 (1.01-1.20)	37.6%	
Facial Deformities				NO
	<20	1.14 (0.99-1.31)	0.0%	
	≥40	1.08 (1.00-1.17)	0.0%	
Musculoskeletal Abnormalities				YES
	<20	1.15 (0.86-1.54)	53.7%	
	≥40	1.19 (0.99-1.42)	41.4%	
Chromosome Disorders				NO
	<20	1.38 (1.01-1.89)	52.6%	
	≥40	1.30 (1.12-1.52)	62.1%	

As for cardiovascular abnormalities, a previous study suggested that older fathers were not a risk factor (OR: 1.15, 95 % CI: 0.96 – 1.36); this finding is consistent with our results [52]. However, the other study was in line with our results in cardiovascular abnormalities (OR: 1.27, 95 % CI: 1.14 – 1.42) [53]. Our meta-analysis controlled for the confounding of maternal age, and we included not only congenital heart defects but also vascular malformations. This might indicate that the difference is due to additional vascular malformations. Conversely, their meta-analysis did not set an age reference group for fathers, whereas our reference group was 25 – 29 years. Two meta-analyses were consistent with our findings that advanced paternal age did not increase the risk of facial deformities; however, these studies focused on orofacial clefts only [52, 54]. Our finding that paternal age was not a risk factor was similar to that of Oldereid [52] (OR: 0.98, 95 % CI: 0.72 – 1.32) in nervous system malformations but different with Jia [55]; the latter reported that younger paternal age (< 20) significantly increased the risk of neural tube defects compared with 25 – 29 years (OR: 1.41 (1.10–1.81)). However, our study encompasses not only neural tube defects but also other neurological diseases, such as hydrocephalus.

The exact mechanism by which young and old paternal age increase the risks of birth defects in offspring is unclear. However, the decline in sperm quality in older men has been demonstrated by several studies, even resulting in infertility. Androgen levels drop significantly in older fathers, and some significant abnormalities in sperm parameters, including the decrease in the total number of Sertoli cells, have been identified in human and animal models [56–59]. Aging could result in testicular histomorphology abnormalities [60], which are the underlying mechanisms of infertility and adverse pregnancy outcomes in older men. Sperms are also associated with an increased abnormal chromosome segregation during meiosis, which may lead to chromosomal defects, including trisomy 21 in progeny [61–63]. Studies have shown that an increase in the number of genetic mutations carried by offspring is related to the age at which the parents conceived [64]. As the father grows older, the number of mutations in the father’s genome increases, leading to an increase in the incidence of congenital malformations in offspring [11, 65].

Older paternal age may be harmful to the offspring’s health in terms of genetic mutations, telomere length, and epigenetics [66]. Several lines of evidence suggest that epigenetic changes occur in the sperm of older fathers, particularly defects in DNA methylation [67–69]. As fathers age, they are exposed to various environmental risk factors, which are involved in the formation and maintenance of epigenetic patterns; these epigenetic modifications have serious consequences for offspring, often contributing to the early onset of diseases [70, 71]. Common environmental risk factors include physical factors (such as radiation and high temperature), chemical factors (such as alcohol, aromatic compounds, heavy metals), and biological factors (such as viruses and bacteria). Older fathers have less antioxidant capacity, and environmental risk factors which may lead to new mutations and DNA damage in some key DNAs related to fetal development [72–74]. Interestingly, a study found that young and old fathers increase the risks of new dominant autosomal mutations, leading to various birth defects in their offspring [75].

A few studies have found that young fathers increase the risk of adverse pregnancy outcomes; unfortunately, fewer studies have focused on the mechanisms. Steiner found that younger fathers have a higher risk of chromosomal aneuploidy in their offspring [76]. Interestingly, Steiner assumed if a 35-year-old woman receives the sperm of a 20-year-old man, her offspring almost doubled their risks of aneuploidy compared with the sperm of a 40-year-old man; meanwhile, the odds of a dominant de novo mutation increased. Moreover, a recent study maintains that young fathers could contribute a substantial load of point mutations to their offspring [77]. Chromosomal aneuploidy and point mutations may partially explain that young fathers increase the risk of some birth defects in newborns, but other factors might be involved, too. The early-bearing population may be at low socioeconomic status [78]. Consequently, factors, including the nutrition of the father or the pregnant woman, and the family's health care affect the health of the fetus in many ways. This phenomenon may be due to unplanned pregnancies among young people, who probably do not take prenatal supplements (such as folic acid) and may be continuously exposed to environmental risk factors (such as smoking) [79–83]. Young fathers may take in additional acrylamide because of special dietary habits and high-temperature food [84]. The metabolites of acrylamide have strong genotoxicity [84], which can indirectly lead to the alkylation of protamine, DNA breakage, and chromosome aberration [85, 86]. Some substances in the semen of young fathers may alter the normal structure and function of sperm, leading to birth defects. Uric acid is highly concentrated in young male semen [87]; such high concentration has been shown to adversely affect sperm morphology and functions [88]. This may be a potential mechanism despite the lack of relevant clinical data validation. Young fathers under stress may contribute to poor birth outcomes, as pre-pregnancy stress may lead to changes in epigenetics [89, 90]. In short, although the exact mechanism is unclear, birth defects caused by young and older fathers may be attributed to some interactions between environmental and genetic factors.

Our findings showed that paternal age, particularly that of young or old fathers is associated with an increased risk of birth defects, indicating that men’s childbearing age should not be too early or too late. Moreover, the implementation of strategic interventions and appropriate preventive measures to reduce the risks of birth defects in offspring are of paramount importance. We found that epidemiological studies in developing countries (especially in Africa, Latin America, and Asia) are relatively few, and future research needs to happen in these regions. Furthermore, some birth defects, such as those of the respiratory, endocrine, and skin systems, have been poorly studied. This requires further study to fill this gap. Future research may also focus on the mechanisms by which paternal age leads to birth defects in offspring for improved prevention and intervention.

### Strengths and limitations

We investigated the influences of old and young paternal age on offspring in this systematic review and meta-analysis. This study neither summarized all birth defects into one category nor analyzed a single birth defect; instead, the study divided them into seven categories according to body regions or systems. The analysis from a systematic perspective is unable to lower the heterogeneity among studies but also incorporate papers about birth defects as many as possible. Moreover, we included some literature published recently in the systematic evaluation. The number of cases of birth defects included in our study was large, with each study sample covering over 5,000 cases, among which the number of cases of cardiovascular abnormalities and musculoskeletal abnormalities exceeded 30,000 cases. Studies that did not control for the confounding of maternal age were excluded, and all high-quality studies (NOS score ≥ 7) were included in the quantitative synthesis, thus improving the reliability of the results to a certain extent. Lastly, this work is the first meta-analysis on the effect of paternal age on urogenital abnormalities in offspring. This can guide clinicians to use some methods before or after delivery, such as ultrasound examination, to check these potential defects in offspring.

However, this systematic review and meta-analysis also have some limitations. Although, tens of studies were retrieved, only a few studies could involve quantitative synthesis as some studies did not control for confounding factors, such as maternal age or different stratification methods of father’s age. Again, most of the estimates and 95 % CI are close to 1.00. For the limitation of the number of articles, we could not perform the subgroup analysis of the countries, years, and specific "isolated" malformations of the study. Although most of the birth defects were included in this meta-analysis, some unusual birth defects, such as respiratory birth defects, were not available. In addition, different studies may have some differences in the definition and classification of birth defects. Some research may only study live births, whereas some studies include birth defects during stillbirth and thus may reduce the accuracy of the results to some extent. The marked etiologic and pathogenic heterogeneity involved in organ systems and body region malformations in this meta-analysis might constitute a major bias regarding the etiologic effect of paternal age. The results showed that heterogeneity was almost minimal (most of I^2^ < 50 %), except for musculoskeletal birth defects and chromosomal abnormalities (Table 2). However, this heterogeneity may not be important because the systematic review and meta-analysis are based exclusively on observational studies and not on intervention trials. We do not circumscribe the meta-analysis to those specific "isolated" malformations but classified them by body regions because the number of studies on specific birth defects is limited.

## CONCLUSIONS

The results of this systematic review and meta-analysis showed that young fathers (< 20 years) could increase risks of urogenital abnormalities and chromosome disorders in their offspring. On the other hand, old fathers (≥ 40 years) could increase risks of cardiovascular abnormalities, facial deformities, urogenital abnormalities, and chromosome disorders in their offspring. In general, younger fathers had less effect on birth defects compared with older ones. Albeit of a moderate effect, we have yet to understand the plausible effects of young or old paternal age in the onset of congenital defects in their offspring. To determine whether paternal age have an adverse effect on specific "isolated" malformations in offsprings, more high-quality prospective cohort studies are needed to be conducted in the future.

## MATERIALS AND METHODS

This study strictly followed the Preferred Reporting Items for Systematic Reviews and Meta-Analyses (PRISMA) criteria. The protocol has been registered with PROSPERO (CRD42020180376).

### Search strategy and selection criteria

We systematically searched original research on Pubmed, Web of Science, the Cochrane Library, and Embase online databases from 1960 to February 2020; references to selected articles were also searched. The retrieval process on PubMed database is as follows: (“Paternal Age”[Mesh] OR (((“male age” OR “man age”) OR “men age”) OR “father age”)) AND (“Congenital Abnormalities”[Mesh] OR ((((((“congenital abnormality” OR “congenital disorder”) OR “congenital disorders”) OR “congenital malformation”) OR “congenital malformations”) OR “birth defect”) OR “birth defects”)). All terms were searched through a combination of the field “Title / Abstract,” but no filters were used to retrieve the literature.

### Inclusion criteria

Observational epidemiologic studies, including cohort and case-control studies published in English; examined the association between paternal age and birth defects in infants; reported ORs and 95 % confidence intervals (CIs) or had raw data available. For multiple publications using the same database, we chose the study that contains the most comprehensive information.

### Exclusion criteria

Studies that were not adjusted or controlled for maternal age, had unclassified birth defects, had no available full text or complete data, and involved animal experiments.

### Outcome measures

This study mainly focuses on urogenital abnormalities, digestive system abnormalities, nervous system malformations, cardiovascular abnormalities, facial deformities, musculoskeletal abnormalities, and chromosome disorders. All these defects have been mentioned in the introduction. Supplementary Table 3 shows the selected birth defects of each study included in the meta-analysis.

### Data extraction and quality assessment

Studies that met the inclusion criteria were independently reviewed by two authors (Y.F. and J.X.), and discrepancies between the authors were resolved through a consensus with a third author (Z.F.). The following information were extracted in a standardized format: first author and year of publication, study period and location, study design, sample size (case/population or case/control), types of birth defects, paternal age categorization, ORs (95 % CI), and adjusted factors.

The methodological quality of the study was evaluated independently by two evaluators (Y.F. and J.X.) in accordance with the Newcastle–Ottawa Quality Assessment Scale (NOS) [91]. NOS has been widely used to evaluate the quality of cohort and case-control studies and strongly recommended by Cochrane. We defined a score of 7–9 as high quality, 5–6 as medium quality, and 0–4 as low quality. The conflicting results were resolved through a discussion between two authors.

### Statistical analysis

To improve reliability, we only included the studies of the reference group with fathers aged 25–29 years into the quantitative meta-analysis. Moreover, studies included in the meta-analysis should be at least adjusted or controlled for maternal age. We quantitatively synthesized the ORs (95 % CI) of each study and compared birth defects in the offspring of young (< 20 years old) and old fathers (> 40 years old) with those of fathers aged 25–29 years old. The results in this study only report random-effect models due to the potential heterogeneity of the study. Funnel plots and Egger’s test were used to assess publication bias, and a nonparametric trim-and-fill method was conducted to correct publication bias. Stata12.0 was used for statistical analysis, and p < 0.05 was considered statistically significant.

## Supplementary Material

Supplementary Figures

Supplementary Table 1

Supplementary Table 2

Supplementary Table 3
