# Supplementary material for: Effect of paternal age on offspring birth defects: a systematic review and meta-analysis
Source: Aging (Albany NY). 2020 Nov 20;12(24):25373–94. doi: 10.18632/aging.104141 (PMC7803514; doi:10.18632/aging.104141)
Supplement: Supplementary Figures [file aging-12-104141-s001.pdf]

SUPPLEMENTARY FIGURES

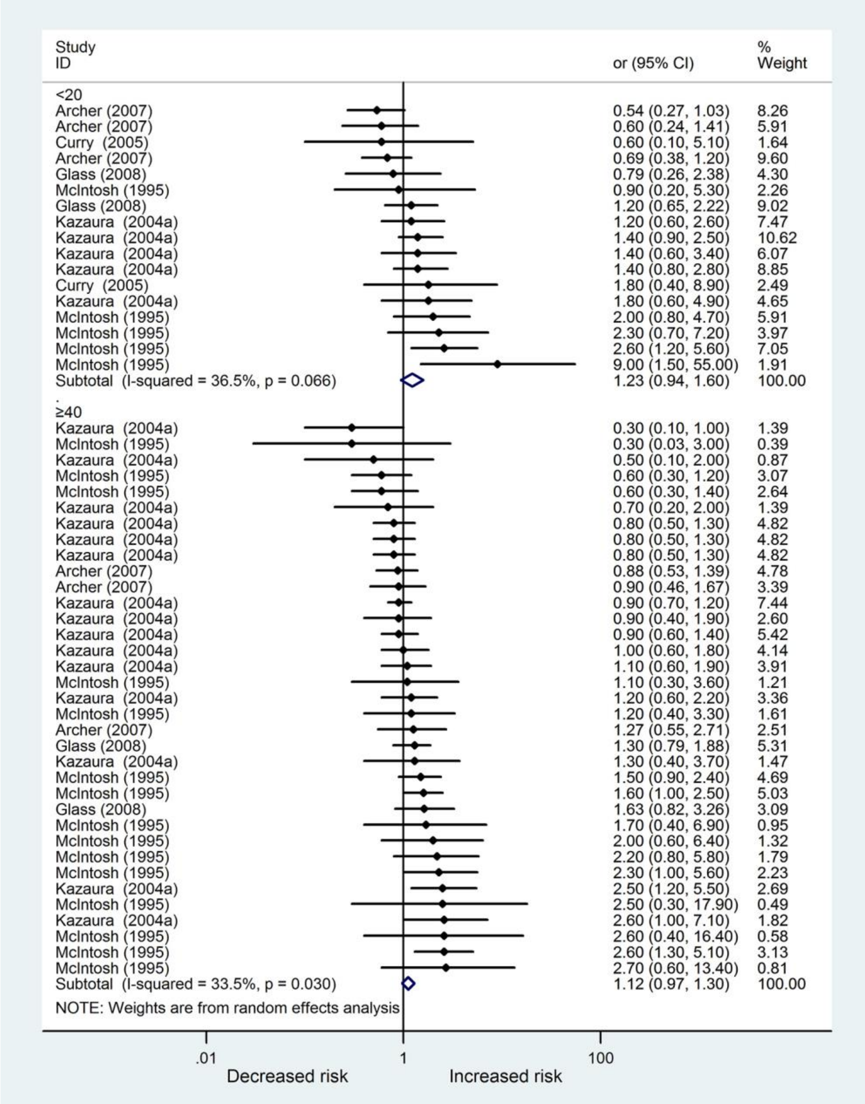

Supplementary Figure 1. Forest plot presenting the effect of young and old father on nervous system malformations in their offspring: Five studies were included in the meta-analysis. The pooled OR in subgroup of young fathers and old fathers was 1.23(95%CI 0.94-1.60) and 1.12(95%CI 0.97-1.30), respectively. There was minimal heterogeneity in these two subgroups (I^2=36.5%, 33.5%, respectively) amongst the studies.

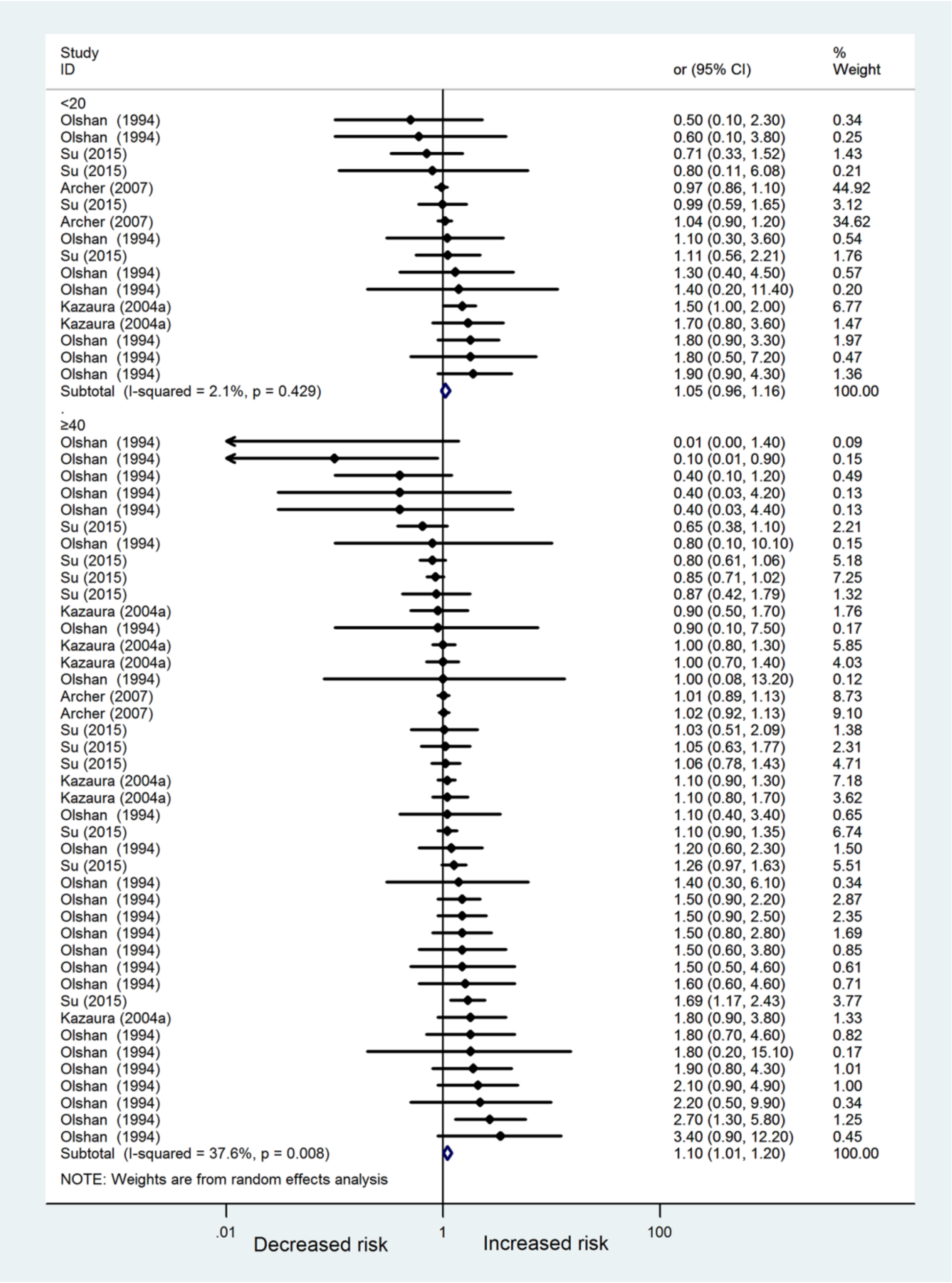

Supplementary Figure 2. Forest plot presenting the effect of young and old father on cardiovascular abnormalities in their offspring: Meta-analysis of the data based on four studies showed that, compared with fathers aged 25 to 29, younger fathers (<20 years) did not increase the risk of cardiovascular abnormalities in their children, while older fathers (≥40 years) did, the pooled OR was 1.05 (95%CI 0.96-1.16) and 1.10 (95%CI 1.01-1.20), respectively. There was minimal heterogeneity (I^2= 2.1%, 37.6%, respectively) amongst the studies.

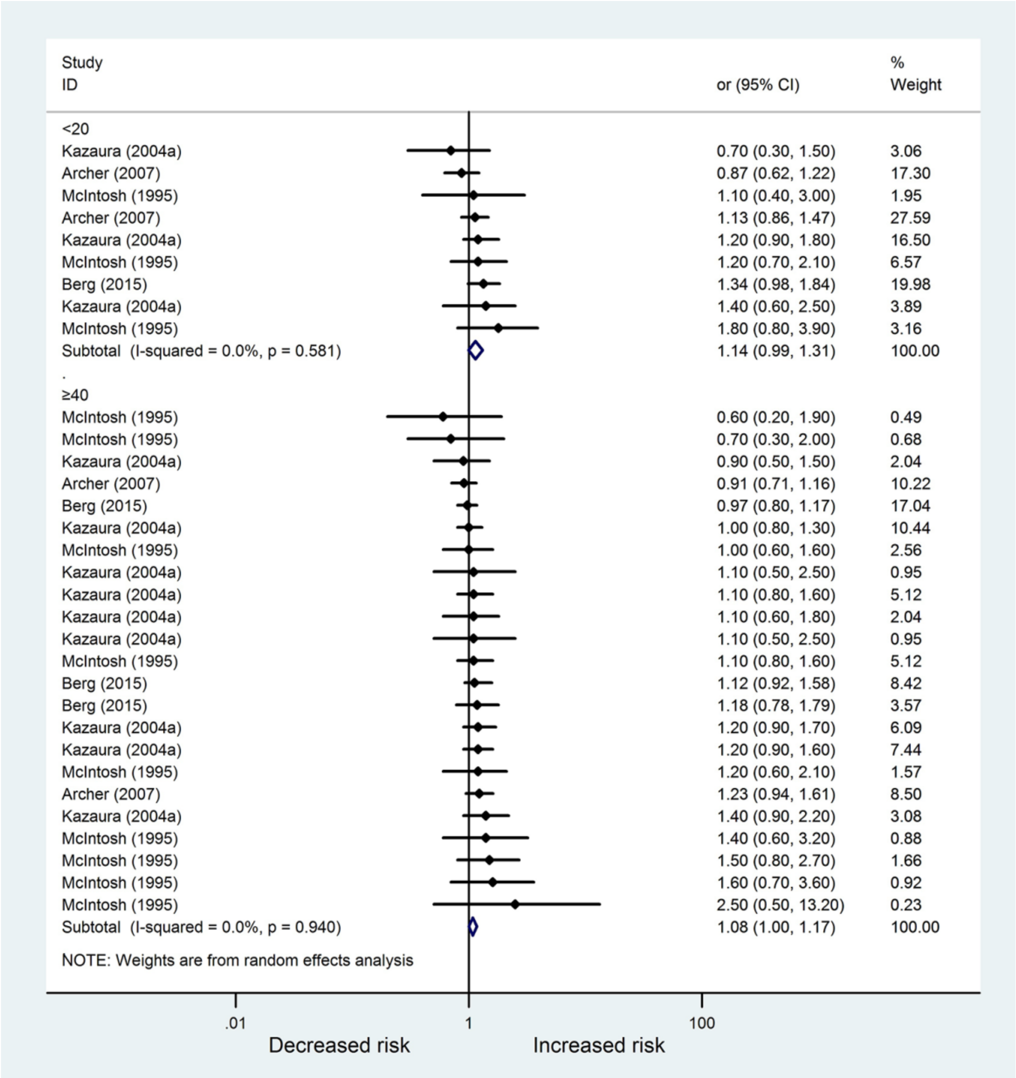

Supplementary Figure 3. Forest plot presenting the effect of young and old father on facial deformities in their offspring: Four studies were included in the meta-analysis. Older fathers (≥40 years) slightly increase the risk of facial deformities in their children, while younger fathers (<20 years) did not, the pooled OR was 1.08 (95%CI 1.00-1.17) and 1.14 (95%CI 0.99-1.31), respectively. There was no heterogeneity in these two subgroups (I^2=0.0%, 0.0%, respectively) amongst the studies.

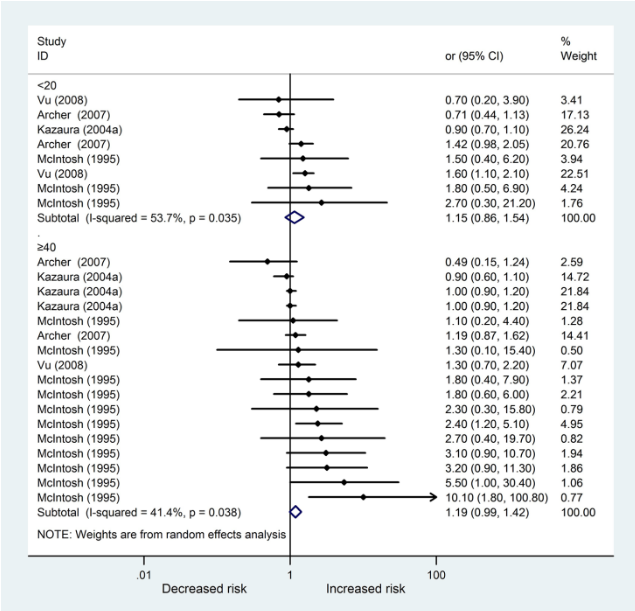

Supplementary Figure 4. Forest plot presenting the effect of young and old father on musculoskeletal abnormalities in their offspring: Five studies were included in the meta-analysis and the results showed that, compared with fathers aged 25 to 29, younger fathers (<20 years) and older fathers (≥40 years) did not increase the risk of musculoskeletal abnormalities in offspring, the pooled OR was 1.15 (95%CI 0.86-1.54) and 1.19 (95%CI 0.99-1.42), respectively. There was medium heterogeneity (I^2= 53.7%, 41.4%, respectively) amongst the studies.

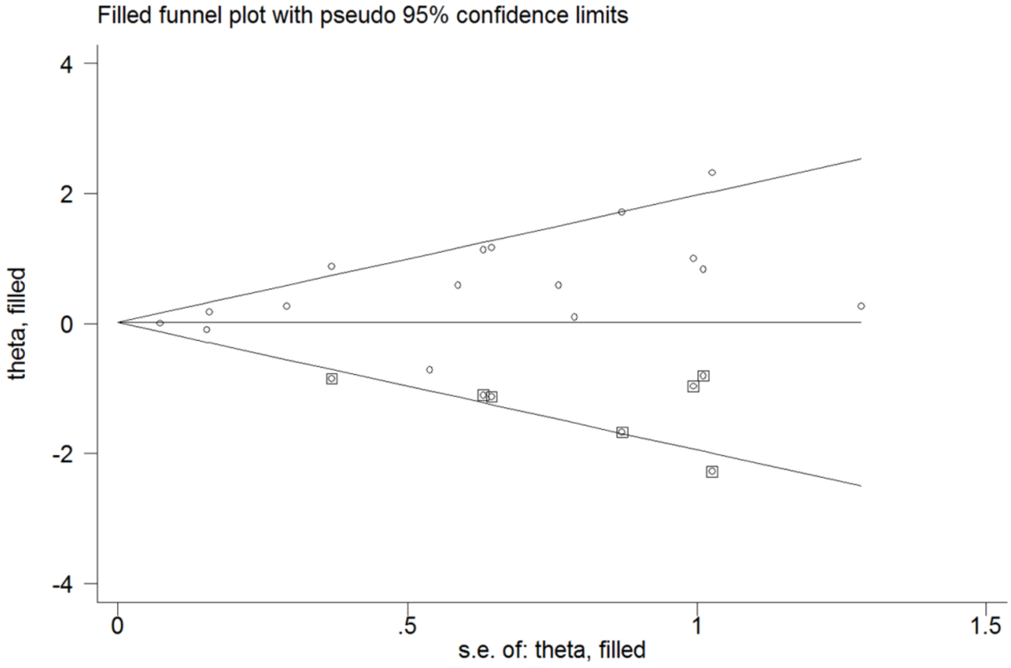

Supplementary Figure 5. The Funnel plots after correcting publication bias in the subgroup of old father by the nonparametric trim and fill method: After correcting publication bias in the subgroup of old fathers by the nonparametric trim and fill method, the pooled OR was still not statistically significant (OR 1.039; 95%CI 0.841-1.284). The dots represent the included studies, and the little square represents complementary studies.

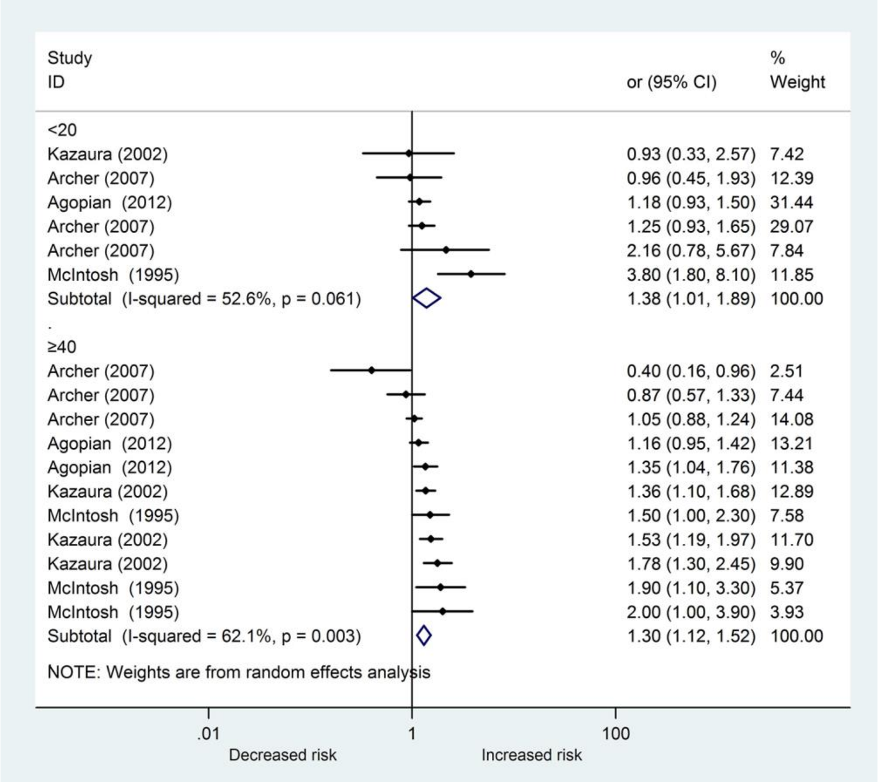

Supplementary Figure 6. Forest plot presenting the effect of young and old father on chromosome disorders in their offspring: Meta-analysis including four studies which showed a moderate high risk of chromosome disorders in newborns of both young and old fathers (OR 1.38, 95%CI 1.01-1.89; OR 1.30, 95%CI 1.12-1.52, respectively), compared with the reference fathers (25-29 years). There was medium heterogeneity (I^2= 52.6%, 62.1%, respectively) amongst the studies.
