# Supplementary material for: Effect of paternal age on offspring birth defects: a systematic review and meta-analysis
Source: Aging (Albany NY). 2020 Nov 20;12(24):25373–94. doi: 10.18632/aging.104141 (PMC7803514; doi:10.18632/aging.104141)
Supplement: Supplementary Table 1 [file aging-12-104141-s002.pdf]

Supplementary Table

Supplementary Table 1. Classification of 7 congenital malformations according to PubMed database.

Classification	Birth defects	Links
Urogenital abnormalities	bladder extrophy, cryptorchidism, disorders of sex development, epispadias, Fraser syndrome, fused kidney, hypospadias, multicystic dysplastic kidney, hereditary nephritis, pyelectasis, and retrocaval ureter	https://www.ncbi.nlm.nih.gov/mesh/68014564
Digestive system abnormalities	anorectal malformations, imperforate anus, biliary atresia and choledochal cyst, diaphragmatic eventration, esophageal atresia, Hirschsprung disease, intestinal atresia, Meckel's diverticulum, and pancreaticobiliary maljunction	https://www.ncbi.nlm.nih.gov/mesh/68004065
Nervous system abnormalities	agenesis of corpus callosum, central nervous system cysts, central nervous system vascular malformations, Dandy–Walker syndrome, hereditary sensory and autonomic neuropathy, hereditary sensory and motor neuropathy, hydranencephaly, malformations of cortical development, neural tube defects, optic nerve hypoplasia, and septo-optic dysplasia	https://www.ncbi.nlm.nih.gov/mesh/68009421
Cardiovascular abnormalities	Congenital heart defects and vascular malformations	https://www.ncbi.nlm.nih.gov/mesh/68018376
Facial deformities	congenital microtia, eye abnormalities, and stomatognathic system abnormalities	https://www.ncbi.nlm.nih.gov/mesh/68000013
Musculoskeletal abnormalities	arthrogryposis, campomelic dysplasia, cervical rib syndrome, craniofacial abnormalities, funnel chest, gastroschisis, Hajdu–Cheney syndrome, congenital hip dislocation, Klippel–Feil syndrome, laryngomalacia, congenital limb deformities, pectus carinatum, synostosis, and tracheobronchomalacia	https://www.ncbi.nlm.nih.gov/mesh/68009139
Chromosome disorders	22q11 deletion syndrome, Angelman syndrome, Beckwith–Wiedemann syndrome, branchiootorenal syndrome, Cri du Chat Syndrome, De Lange syndrome, Down syndrome, holoprosencephaly, Jacobsen distal 11q deletion syndrome, Prader–Willi syndrome, Rubinstein–Taybi syndrome, sex chromosome disorders, Silver–Russell syndrome, Smith–Magenis syndrome, Sotos syndrome, Trisomy 13 syndrome, Trisomy 18 syndrome, WAGR syndrome, Williams syndrome, and Wolf–Hirschhorn syndrome	https://www.ncbi.nlm.nih.gov/mesh/68025063
