# Supplementary material for: Effect of paternal age on offspring birth defects: a systematic review and meta-analysis
Source: Aging (Albany NY). 2020 Nov 20;12(24):25373–94. doi: 10.18632/aging.104141 (PMC7803514; doi:10.18632/aging.104141)
Supplement: Supplementary Table 2 [file aging-12-104141-s003.docx]

Supplementary Table 2. The characteristics of the included studies in the systematic review.

| Author, year | Study period and location | Study design | Defects: number of cases | Paternal age categorization | Influence of early age on birth defects | Influence of older age on birth defects | Adjust/ Control for maternal age | NOS |
| --- | --- | --- | --- | --- | --- | --- | --- | --- |
| Abqari (2016) | 2014-2015, India | Case-control | CHDs: 400 | **<25,** >25 | NO | YES. Increased | Without adjusted | 5 |
| Agopian (2012) | 1999-2007, USA | Cohort | Trisomy 21: 4117 | <20, 20–24, **25-29**,30–34, 35–39, ≥40 | NO | YES. Increased | Control for maternal age. | 7 |
| Archer (2007) | 1996–2002, USA | Cohort | Anencephaly: 248; Spina bifida: 564; Encephalocele: 118; Ventricular septal defect: 6790; Atrial septal defects: 6403; Cleft palate alone: 920; Cleft lip ± cleft palate: 1657; Pyloric stenosis: 3007; Craniosynostosis: 636; Gastroschisis: 550; Trisomy 21: 1870; Trisomy 13: 143; Trisomy 18: 278 | <20, 20–24, **25-29**, 30–34, 35–39, ≥40 | NO | YES. Decreased: Pyloric stenosis, Trisomy 13 | Adjusted for maternal age, et al. | 7 |
| Avilés (2014) | 2007-2010, USA | Case-control | hypospadias: 279 | <20, 20–24, **25-29**,30–34, 35–39, 40-44, ≥45 | NO | NO | Without adjusted. | 6 |
| Barbosa-Buck (2012) | 2000-2007, Latin America | Case-control | Skeletal Dysplasias: 472 | **≤39**, >39 | - | YES. Increased | Without adjusted. | 4 |
| Bassili (2000) | 1995-1997, Egypt | Case-control | CHDs: 894 | ≤20, **21-40**, >40 | YES. Decreased | YES. Increased | Adjusted for maternal age, et al. | 8 |
| Berg (2015) | 1967-2010, Norway | Cohort | cleft lip: 2890 | <20, 20–24, **25-29**,30–34, 35–39, 40-44, 45-49, ≥50 | NO | NO | Adjusted for maternal age, et al. | 9 |
| Bille (2015) | 1973-1996, Denmark | Cohort | cleft lip ± cleft palate: 1920; cleft palate only: 956 | Not categorized | NO | YES. Increased | Adjusted for maternal age. | 8 |
| Cedergren (2002) | 1982–1996, Sweden | Case-control | CHDs: 269 | ≤19, 20–24, **25-29**,30–34, 35–39, 40-44, ≥45 | NO | NO | Without adjusted. | 7 |
| Chabra (2011) | 1987–2006, USA | Cohort | Gastroschisis: 282 | ≤19, 20–24, **≥25** | YES. Increased | NO | Adjusted for maternal age, et al. | 9 |
| Corona-Rivera (2019) | 2009-2017, Mexico | Case-control | Down syndrome: 211 | ≤19, 20–24, 25-29,30–34, 35–39, ≥40 | YES. Increased | NO | Adjusted for maternal age, et al. | 8 |
| Curry (2005) | 1985-2001, USA | Cohort | Isolated Schizencephaly: 30; Non-Isolated Schizencephaly: 19 | ≤19, 20–24, **25-29**,30–34, ≥35 | NO | NO | Adjusted for maternal age, et al. | 8 |
| De Souza (2009) | 1960s, UK | Case-control | Trisomy 21: 471 | Not categorized | NO | NO | Adjusted for maternal age. | 5 |
| De Souza (2010) | 1980-2005, EU | Case-control | Trisomy 21: 5627; Trisomy 13: 374; Trisomy 18: 929; Klinefelter: 295; XYY: 28 | Not categorized | NO | YES. Increased: Trisomy 21, Klinefelter | Controls were matched on maternal age | 7 |
| Downing (2010) | 1997-2011, USA | case–control | Ebstein anomaly: 135 | <20, **20–34**, 35–39, ≥40 | NO | YES. Increased | Adjusted for maternal age, et al. | 9 |
| Dzurova (2005) | 1996-1997, USA; 1994-1998 Czech | Cohort | Down syndrome USA: 593; the Czech: 251 | **≤19**, 20–22, 23–25, 26–28, 29–31, 32–34, 35–37, 38–40, 41–43, ≥44 | NO | NO | Adjusted for maternal age, et al. | 9 |
| Glass (2008) | 1983-2003, USA | Cohort | Agenesis of the Corpus Callosum: 472  Hypoplasia of the Corpus Callosum: 158 | <20, 20–24, **25–29**, 30–34, 35–39, 40-55 | NO | NO | Adjusted for maternal age. | 8 |
| Harville (2007) | 1967-1998, Norway | Cohort | Cleft palate, no accompanying defect: 893; Cleft palate, accompanying defect: 435 | <20, **20–24**, 25-29, 30–34, 35–39, ≥40 | YES. Increased: Cleft palate, no accompanying defect | NO | Stratified for maternal age,  20–29 years | 7 |
| Kazaura (2004a) | 1967-1998, Norway | Cohort | Neural tube defects: 1303; Anencephaly: 512; Spina bifida: 791; Hydrocephaly: 583; Other CNS: 322; Ear/Face/Neck: 950; Heart: 3656; Circulatory: 764; Isolated cleft: palate 883; Total cleft lip: 2354; Anal: 822; Renal: 1061; Limb: 11255 | <20, 20–24, **25-29**, 30–34, 35–39, 40-44, 45-49, ≥50 | YES. Increased: Heart | YES. Increased: Other CNS | Adjusted for maternal age, et al. | 8 |
| Kazaura (2004b) | 1967-1998, Norway | Cohort | Gastroschisis: 291 | Not categorized | YES. Increased | NO | Adjusted for maternal age, et al. | 8 |
| Kazaura (2002) | 1967-1998, Norway | Cohort | Down’s syndrome: 1788 | <20, 20–24, **25-29**, 30–34, 35–39, 40-44, 45-49, ≥50 | NO | YES. Increased | Adjusted for maternal age, et al. | 8 |
| Liu (2018) | 2014-2017, China | Case control | microtia/atresia: 322 | **<26**, 26–30, 31–35, ＞35 | NO | YES. Increased | Without adjusted | 7 |
| Martelli (2010) | 2006-2008, Brazil | Case control | cleft lip-palate: 100 | **≤40**, > 40 | NO | YES. Increased | Without adjusted | 8 |
| Materna-Kiryluk (2009) | 1998-2002, Poland | Cohort | Neural tube defects:599; Microcephaly:58; Hydrocephaly:202; CHDs: 3933; Cleft palate:412; Cleft lip±cleft palate:745; Oesophageal atresia:105; intestine atresia or stenosis:123; Ano-rectal atresia or stenosis: 87; Hypospadias: 991; Renal agenesis or Hypoplasia: 124; Cystic kidney disease:167; Congenital hydronephrosis:177; Diaphragmatic hernia: 86; Omphalocele:69 Gastroschisis:120 | Not categorized | YES. Increased: Gastroschisis | YES. Increased: Congenital heart defects, Cleft palate, Cleft lip ± cleft palate, Hypospadias, | Adjusted for maternal age | 8 |
| McIntosh (1995) | 1952-1973, Canada | Case control | Neural tube defects: 713; Anencephaly: 127; Spina bifida: 595; Microcephaly: 154 Hydrocephaly: 384; Congenital cataracts: 300; Cleft palate: 514; Cleft lip ± palate: 1376; Tracheoesophageal fistula: 85; Pyloric stenosis: 855; Atresia of the intestine: 191; Hirschsprung’s disease: 84; Hypospadias: 854; Renal agenesis: 131; Cystic kidney: 80; Obstructive renal defects: 299; Atresia of the urethra: 421; Reduction defect of upper limb: 352; Reduction defect of lower limb: 130; Chondrodystrophy: 92; Anomaly of the diaphragm: 117; Down syndrome: 969 | <20, 20–24, **25-29**, 30–34, 35–39, 40-44, 45-49, ≥50 | YES. Increased: Neural tube defects, Anencephaly, Hypospadias,  Down syndrome | YES. Increased: Hydrocephaly (40-44), Hypospadias (45-49), Chondrodystrophy (45-49), Down syndrome | Adjusted for maternal age, et al. | 8 |
| Naguib (1999) | 1980-1997, Kuwait | Case control | Trisomy 18: 118 | **<30**, 30–34, 35–39, 40-44, 45-49, ≥50 | NO | NO | Adjusted for maternal age, et al. | 6 |
| Olshan (1994) | 1952-1973, Canada | Case control | Ventricular septal defects: 1055; Atrial septal defects: 646; Patent ductus arteriosus: 457; Coarctation of aorta: 173; Pulmonary artery anomalies: 235; Tetralogy of Fallot: 195; Transposition of great vessels: 147; Pulmonary valve anomalies: 171 | <20, 20–24, **25-29**, 30–34, 35–39, 40-44, 45-49, ≥50 | NO | YES. Increased:  Atrial septal defects (45-49) Decreased: Pulmonary artery anomalies (45-49) | Adjusted for maternal age, et al. | 8 |
| Orioli (1995) | 1967-1992, Italy | Case control | Achondroplasia: 31; Thanatophoric dysplasia: 34; Osteogenesis imperfecta: 60 | **＜35**, ＞35 | NO | NO | Without adjusted | 6 |
| Riccardi (1984) | 1978-1983, USA | Case control | Neurofibromatosis: 58 | **＜35**, ＞35 | NO | YES. Increased | Controlled for maternal age | 5 |
| Savitz (1991) | 1959-1966  USA | Cohort | Nasal aplasia: 23; Hydrocephalus: 18; Ventricular septal defect: 47; Hypospadias: 56; Polydactyly: 55; Hemangioma: 20 | **<30**, 30–34, 35–39, ≥40 | NO | YES. Increased: Hydrocephalus, Hemangioma | Adjusted for maternal age, et al. | 7 |
| Su (2015) | 1977-2008, Denmark | Cohort | PDA: 1748; ASD: 2543; VSD: 3628; TOF: 365; COA: 458 | <20, 20–24, **25-29**, 30–34, 35–39, 40-44, ≥45 | NO | YES Increased: PDA (≥45) | Adjusted for maternal age, et al. | 8 |
| Tufekci (2018) | ---, Turkey | Case control | Cleft lip ± palate: 42 | ＜30, ＞30 | NO | NO | Without adjusted | 4 |
| Urhoj (2015) | 1978-2004, Denmark | cohort | Musculoskeletal Congenital Anomalies: 10817 | <25, 25-29, **30–34**, 35–39, 40-44, 45-49, ≥50 | YES. Decreased | YES. Decreased (≥50) | Adjusted for maternal age, et al. | 8 |
| Vu (2008) | 1987-2003, USA | cohort | Gastroschisis: 835 | 12-15, 16-19, 20-24, 25-29, 30-34, 35-39, 40-90 | NO | NO | Adjusted for maternal age, et al. | 8 |
| Waller (2008) | ---, USA | cohort | Achondroplasia: 79 | **<25**, 25-29, 30–34, 35–39, ≥40 | NO | YES. Decreased | Without adjusted | 6 |
| Yun (2016) | 2006, China | Cohort | Cleft Palate: 215 | Not categorized | NO | YES. Decreased | Without adjusted | 6 |
| Zhan (1991) | 1998, China | Case control | congenital heart disease: 497 | ＜25, **＞25** | YES. Decreased | NO | Without adjusted | 5 |
| Zhu (2005) | 1980-1996, Denmark | Cohort | Nervous system: 163; Eye, ear, face and neck: 738; Circulatory system: 494; Cleft lip/palate: 162; Digestive system: 464; Genital organs: 390; Urinary system: 187; Extremities: 940; Musculoskeletal system: 243; Down’s syndrome: 46 | **20-29**, 30–34, 35–39, 40-44, 45-49, ≥50 | NO | YES. Decreased: Extremities (40-49), Down’s syndrome (≥50) | Adjusted for maternal age, et al. | 9 |
