# Supplementary material for: Effect of paternal age on offspring birth defects: a systematic review and meta-analysis
Source: Aging (Albany NY). 2020 Nov 20;12(24):25373–94. doi: 10.18632/aging.104141 (PMC7803514; doi:10.18632/aging.104141)
Supplement: Supplementary Table 3 [file aging-12-104141-s004.pdf]

Supplementary Table

Supplementary Table 3. Selected birth defects of each study included in meta-analysis.

Classification	Birth defects	Reference
Urogenital Abnormalities	birth defect in renal	Kazaura (2004a)
	Renal agenesis	McIntosh (1995)
	Cystic kidney	McIntosh (1995)
	Obstructive renal defects	McIntosh (1995)
	Hypospadias	McIntosh (1995)
	Atresia of the urethra	McIntosh (1995)
Digestive System Abnormalities	Pyloric stenosis	Archer (2007), McIntosh (1995)
	Anal	Kazaura (2004a)
	Tracheoesophageal fistula	McIntosh (1995)
	Atresia of the intestine	McIntosh (1995)
	Hirschsprung's disease	McIntosh (1995)
Nervous System Malformations	Anencephaly	Archer (2007), Kazaura (2004a), McIntosh (1995)
	Spina bifida	Archer (2007), Kazaura (2004a), McIntosh (1995)
	Encephalocele	Archer (2007)
	Isolated Schizencephaly	Curry (2005)
	Non-Isolated Schizencephaly	Curry (2005)
	Agenesis of the Corpus Callosum	Glass (2008)
	Hypoplasia of the Corpus Callosum	Glass (2008)
	Neural tube defects	Kazaura (2004a), McIntosh (1995)
	Hydrocephaly	Kazaura (2004a), McIntosh (1995)
	Microcephaly	McIntosh (1995)
	Other CNS	Kazaura (2004a)
Cardiovascular Abnormalities	Ventricular septal defect	Archer (2007), Olshan (1994), Su (2015)
	Atrial septal defects	Archer (2007), Olshan (1994), Su (2015)
	Patent ductus arteriosus	Olshan (1994), Su (2015)
	Coarctation of aorta	Olshan (1994), Su (2015)
	Pulmonary artery anomalies	Olshan (1994)
	Tetralogy of Fallot	Olshan (1994), Su (2015)
	Transposition of great vessels	Olshan (1994)
	Pulmonary valve anomalies	Olshan (1994)
	CHDs	Cedergren (2002), Kazaura (2004a)
	Circulatory	Kazaura (2004a)
Facial Deformities	Cleft palate alone, Cleft lip ± cleft palate	Archer (2007), Berg (2015), Kazaura (2004a), McIntosh (1995)
	Ear/Face/Neck	Kazaura (2004a)
	Congenital cataracts	McIntosh (1995)
Musculoskeletal Abnormalities	Craniosynostosis	Archer (2007)
	Gastroschisis	Archer (2007), Vu (2008)
	Limb, Reduction defect of upper limb, Reduction defect of upper limb	Kazaura (2004a), McIntosh (1995)
	Chondrodystrophy	McIntosh (1995)
	Anomaly of the diaphragm	McIntosh (1995)
Chromosome Disorders	Trisomy 21	Agopian (2012), Kazaura (2002), McIntosh (1995)
	Trisomy 13	Archer (2007)
	Trisomy 18	Archer (2007)
